# Intermittent hypoxia reduces infarct size in rats with acute myocardial infarction: a systematic review and meta-analysis

**DOI:** 10.1186/s12872-020-01702-y

**Published:** 2020-09-22

**Authors:** Ke Hu, Wei Deng, Jing Yang, Yu Wei, Chaolin Wen, Xingsheng Li, Qingwei Chen, Dazhi Ke, Guiqiong Li

**Affiliations:** 1 Department of Nephrology, The Second Affiliated Hospital of Chongqing Medical University, No. 74, Linjiang Road, Yuzhong District, Chongqing, 400010 China; 2 Department of Geriatrics, The Second Affiliated Hospital of Chongqing Medical University, No. 74, Linjiang Road, Yuzhong District, Chongqing, 400010 China; 3 Department of Neurology, The Second Affiliated Hospital of Chongqing Medical University, No. 74, Linjiang Road, Yuzhong District, Chongqing, 400010 China

## Abstract

**Background:**

To determine whether intermittent hypoxia (IH) can reduce the infarct size (IS) after acute myocardial infarction (AMI) in rats.

**Methods:**

Articles were identified in PubMed, EMBASE and the Web of Science and were included if they evaluated the effect of IH on the changes in the infarcted area after AMI in rats.

**Results:**

A preliminary search identified 3633 articles and 29 data sets from 23 articles (12 in vivo, 16 in vitro). The IS decreased after AMI in IH rats both in vitro (SMD -1.46, 95% CI [− 2.37, − 0.55]; I^2^ = 85.6%, *P* = 0.000) and in vivo (SMD -1.43, 95% CI [− 2.05, − 0.82], I^2^ = 73.6%, *P* = 0.000). Sensitivity analysis indicated that IH had a strong protective effect against myocardial infarction, and the hypoxia concentration was significantly correlated with the change in IS after AMI.

**Conclusion:**

IH can reduce IS after AMI in rats. This effect of IH may be related to the dose of hypoxia, and the oxygen concentration may be one of the most important influencing factors.

## Background

Intermittent hypoxia (IH) involves inducing the body to continuously self-regulate by repeatedly exposing the subject to a hypoxic or hypobaric hypoxia environment in a certain way [1, 2]. To construct IH animal models, experimental animals such as rats or mice are repeatedly exposed to a hypoxic environment with controlled parameters (including oxygen concentration, air pressure, daily exposure time, total duration, and mode of action). The IH animals are then subjected to coronary artery ligation (20–30 min) in vivo or in vitro. Next, the coronary artery is reperfused for 2–4 h. Finally, the myocardial infarct area of the rats is observed and compared with a normoxic group [3, 4].

An increasing number of studies show that IH can help reduce the area of myocardial infarction in patients with coronary heart disease [5–7] or in animal models of acute myocardial infarction [8, 9]. IH can not only reduce IS but also increase the ejection fraction of the heart and reduce the occurrence of arrhythmia after AMI [8, 10, 11], IH also has protective effects on other organs of the body, such as lowering blood pressure, improving glucose tolerance, improving blood lipid levels, reducing the infarct area after acute cerebral infarction, and improving cognitive dysfunction and renal fibrosis after ischemia [1, 2, 12–15].

However, researchers also found that IH sometimes increases myocardial IS after AMI in rats [16, 17]. Therefore, we conducted a comprehensive systematic review and meta-analysis to evaluate whether the overall effect of intermittent hypoxia on the heart increases or decreases the IS. Multiple subgroup analyses were also performed to further explore how the details of the IH protocol influence the findings. At the same time, this meta-analysis assesses how the robustness of the data analysis and the quality and publication bias of the literature included in the study will impact the findings and overall conclusions.

## Methods

### Literature search

The systematic review was conducted according to the Preferred Reporting Items for Systematic Reviews and Meta-Analyses (PRISMA) guidelines [18]. Ke Hu and Jing Yang performed a literature search of the EMBASE, Medline (PubMed) and Web of Science databases using selected keywords (Heart, Myocardial Infarction, Myocardial Ischemia, Coronary Artery Disease, Myocardial Reperfusion Injury, Myocardium, Hypoxia, Oxygen Deficiency, Altitude, Rat) and Medical Subject Headings (MeSH) terms specific to each database.

The search included literature that investigated the effect of IH on infarct size in either in vivo (in which the coronary artery is occluded in living anesthetized rat hearts) and in vitro (in which the coronary artery is occluded in isolated rat hearts) models after AMI. Searches were performed on October 31, 2019, and included only studies that were available in English. The inclusion criteria were developed in accordance with the PICOS (population, intervention, comparison, outcome, study design) approach [19]. In vivo and in vitro studies were included, and the oxygen concentration, daily duration and total time of IH should also be described in detail. The duration of ischemia and reperfusion should also be recorded.

The following classes of studies were excluded: studies that used persistent IH; studies in which there was no documented reperfusion phase or the coronary artery occlusion was permanent; studies that did not report absolute myocardial IS as a percentage of ventricle size (VS) or area at risk (AAR, defined as the myocardial tissue within the vascular territory that is distal to the occluded artery and, if not reperfused, is at risk of irreversible ischemic death); studies employing genetically modified animals or animals with comorbidity, such as diabetes, heart failure, or high blood pressure; and experimental studies where an IH was administered concomitantly with any other pharmacological treatment, whether it is known for its cardioprotective properties or not.

### Data extraction and quality assessment

Data were independently extracted by two authors (Yu Wei and Chaoling Wen) using a predesigned table, including first author’s name, year of publication, species, age, sex, hypoxic conditions, ligation of coronary artery, duration of index ischemia, reperfusion duration, induction anesthetic, measurement of IS, sample size, infarct size and variance. Disagreements were resolved by consensus in all cases. Whenever key information was missing, we contacted the report authors by e-mail and requested it. We characterized the quality of reporting in the included studies using a predefined 20-point scoring scale based on the Animal Research: Reporting In Vivo Experiments (ARRIVE) guidelines [20, 21] and a 9-point document quality scoring scale based on the Collaborative Approach to Meta-Analysis and Review of Animal Data from Experimental Studies (CAMARADES) list [22, 23]. This evaluation was carried out by Ke Hu and Jing Yang independently. Disagreements were resolved by examining the full text of the article or by reaching consensus between reviewers in all cases.

### Statistical analysis

Stata (version 14.0) was used for all statistical analyses. Whenever outcomes in infarct size were reported on different measurement scales, our primary outcome was expressed as standardized mean difference (SMD) between both experimental groups. The final effect values were expressed as a raw difference in the mean IS/VS% or IS/AAR% (the mean of the control groups minus the mean of the experimental group) and the corresponding 95% confidence intervals (CI). The Q test was used to assess the magnitude of the heterogeneity between studies, with values *p* < 0.1 or I^2^ > 50% taken to indicate a moderate-to-high degree of heterogeneity. When I^2^ is greater than 50%, a random effect model is used; otherwise, a fixed effect model is employed.

Sensitivity analysis was tested by conducting an additional stratified meta-analysis using the unstandardized mean difference (WMD). Furthermore, subgroup analyses based on predefined experimental factors were also performed.

We assessed the possibility of publication bias using Begg’s test and defined significant publication bias as a *P* value < 0.1. A trim-and-fill computation was used to estimate the effect of publication bias on the interpretation of the results.

## Results

### Study selection process

**Fig. 1:**
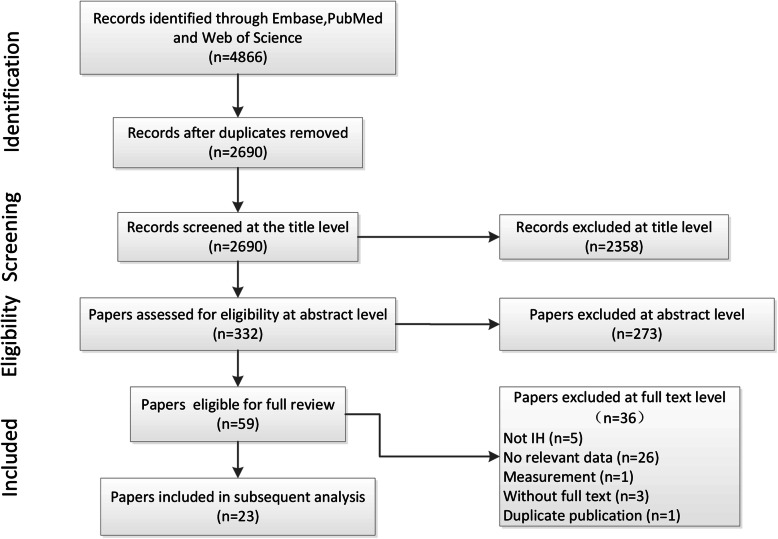
Flow chart of the study selection process

Our preliminary search of three databases identified 4866 documents. After removing 2176 duplicates, we then removed 2358 articles based on their titles, after which 332 articles entered the summary screening stage and 59 articles entered the full-text screening. Ultimately, 23 papers including a total of 28 comparisons were collected. The data were divided into an in vivo group and an in vitro group (Fig. 1, Table 1).

**Table 1:** Summary of the main characteristics of included studies

1	2	3	4	5	6	7	8	9	10	11	12	13	14	15	16	17	18	19
Yuan 2017 [4]	SD	M	330–350 g	5000 m altitude	6 h/day	28 days	G	30	120	sodium pentobarbital	IR/VR	Means±SD	4	12.8	2.0	4	38.4	5
Li 2016 [24]	SD	M	220 ±? g	5000 m altitude	6 h/day	28 days	G	30	120	sodium pentobarbital	IR/VR	Means±SD	6	27.3	5.9	6	48.3	7.5
Kasparova 2015 [3]	W	M	300–380 g	Normobaric FiO2 10%	8 h/day	21 days	LAD	20	180	Sodium pentobarbital	IR/AAR	Means±SE	10	46.4	4.2	10	62.7	2.7
Meng 2014 [25]	W	M	150–180 g	5000 m altitude	6 h/day	42 days	LAD	30	240	25% ethylurethanm solution	IR/AAR	Means±SE	7	12.5	1.6	7	30.9	6.8
Ma 2014 [26]	SD	M	neonate	3000 m altitude	5 h/day	42 days	G	30	120	sodium pentobarbital	IR/VR	Means±SE	6	26.7	0.4	6	48.6	2.2
Gao 2014 [27]	SD	M	100–130 g	5000 m altitude	4 h/day	28 days	G	30	120	sodium pentobarbital	IR/AAR	Means±SE	5	22.58	3	5	35.9	4.1
Manukhina 2013 [28]	W	M	250–280 g	Normobaric FiO2 9.5–10%	4 min/cycle	5–8 cycles/day 20 days	LAD	30	120	urethane	IR/AAR	Means±SE	10	17.5	2.6	9	30.6	5.1
Ramond 2013a [16]	W	M	250–350 g	Normobaric FiO2 5%	40s/min 8 h/d	14 days	G	30	120	sodium pentobarbital	IR/VR	Means±SE	7	36.0	2.8	7	21.8	3.1
Ramond 2013b [16]	W	M	250–350 g	Normobaric FiO2 5%	40s/min 8 h/d	14 days	G	30	120	sodium pentobarbital	IR/VR	Means±SE	8	40.3	3.5	8	29.4	3.7
Milano 2011 [17]	SD	M	251 ± 2 g	Normobaric FiO2 10%	23 h/day	15 days	LAD	30	180	sodium pentobarbital	IR/AAR	Means±SE	7	68.5	2.4	7	46.5	4.4
Wang 2011 [29]	SD	M	100–120 g	5000 m altitude	4 h/day	28 days	G	30	120	pentobarbital sodium	IR/AAR	Means±SE	5	20.5	5.3	5	42.1	3.8
Belaidi 2008 [30]	W	M	330–350 g	Normobaric FiO2 10%	4 h/day	1 day	G	30	120	pentobarbital sodium	IR/VR	Means±SE	8	15.9	5.6	8	33.8	5
Yeung 2007a [31]	SD	M	290–320 g	Normobaric FiO2 10%	6 h/day	3 days	LCA	30	120	decapitated	IR/AAR	Means±SE	6	32.7	1.2	6	35.1	1.8
Yeung 2007b [31]	SD	M	290–320 g	Normobaric FiO2 10%	6 h/day	7 days	LCA	30	120	decapitated	IR/AAR	Means±SE	6	26.5	0.7	6	34.8	1.1
Yeung 2007c [31]	SD	M	290–320 g	Normobaric FiO2 10%	6 h/day	14 days	LCA	30	120	decapitated	IR/AAR	Means±SE	6	31.71	1.0	6	37.7	2.0
Ravingerová 2007 [32]	W	M	250–280 g	7000 m altitude	8 h/day	5 days/week 25–30 days	LAD	20	180	sodium pentobarbiton	IR/AAR	Means±SE	8	51.8	4.4	8	64.9	5.1
Kolár 2007 [33]	W	M	250–280 g	7000 m altitude	8 h/day	24–30 days	LAD	20	180	sodium pentobarbital	IR/AAR	Means±SE	8	27.7	4.9	8	56.7	4.5
Béguin 2007 [34]	W	M	330–380 g	5000 m altitude	4 h/d	1 day	G	30	120	sodium pentobarbital	IR/VR	Means±SE	8	22.2	2.4	8	33.8	2.6
Zhu 2006 [35]	SD	M	100–130 g	5000 m altitude	6 h/day	42 days	G	30	120	sodium pentobarbital	IR/VR (LV area)	Means±SE	7	24	1.9	7	42.1	2.1
Kolár 2005 [36]	W	M	5–6 weeks	7000 m altitude	8 h/day	35–42 days	LAD	20	180	sodium pentobarbiton	IR/AAR	Means±SE	7	43.2	3.3	7	59.2	4.4
Béguin 2005 [37]	W	M	330–380 g	Normobaric FiO2 10%	40s/min 4 h/d	1d	G	30	120	sodium pentobarbiton	IR/VR (LV area)	Means±SE	11	21.8	3.1	9	33.5	2.5
Joyeux-Faure 2005 [38]	W	M	220–240 g	Normobaric FiO2 5%	40s/min 8 h/d	35 days	G	30	120	sodium pentobarbiton	IR/VR	Means±SE	9	46.9	3.6	9	26.1	2.8
Neckár 2005a [39]	W	M	250–280 g	7000 m altitude	8 h/day	24–32 days	LAD	20	180	sodium pentobarbital	IR/AAR	Means±SE	9	41.2	3.9	9	58.2	2.2
Neckár 2005b [39]	W	M	250–280 g	7000 m altitude	8 h/day	24–32 days	LAD	20	180	sodium pentobarbital	IR/AAR	Means±SE	9	33.9	3.8	9	58.9	3.7
Neckár 2004 [40]	W	M	250–280 g	7000 m altitude	8 h/day	5 days a week 35 days	LAD	30	240	sodium pentobarbiton	IR/AAR	Means±SE	11	48	2.2	15	69.2	1.7
Neckár 2002a [41]	W	M	250–280 g	5000 m altitude	8 h/day	5 days a week 24–32 days	G	20	240	sodium pentobarbiton	IR/AAR	Means±SE	16	52.7	2.5	10	62.2	2
Neckár 2002b [41]	W	M	250–280 g	5000 m altitude	8 h/day	5 days a week 24–32 days	G	20	240	sodium pentobarbiton	IR/AAR	Means±SE	9	51.5	2.2	9	60.2	2.6
Neckár 2002c [42]	W	M	250–280 g	7000 m altitude	8 h/day	5 days a week 24–30 days	LAD	30	240	sodium pentobarbiton	IR/AAR	Means±SE	19	50.2	1.9	10	66.6	2.3

Different letters refer to different studies and experimental groups within each included study. SD, Sprague-Dawley rats; W, Wistar rats; M, male; 7000 m altitude (around PB 308 mmHg, PO2 65 mmHg, FiO2 9.75%), 5000 m altitude (around PB 404 mmHg, PO2 85 mmHg, FiO2 12.95%), 3000 m altitude (around PB 525 mmHg, PO2 110 mmHg, FiO2 14.4%), sea-level (around PB 760 mmHg, PO2 159 mmHg, FiO2 20.95%); G, global ischemia; LAD, left anterior descending; LCA, left coronary artery; IS, infarct size; VS, ventricle size; AAR, area at risk; SEM, standard error of the mean; STD, standard deviation of the mean. The main characteristics included (1) study, year, reference; (2) species; (3) sex; (4) age or weight; (5) oxygen concentration (Fi O2); (6) daily hypoxic exposure (hour); (7) total stimulus duration (day); (8) ligation of coronary artery; (9) duration of index ischemia duration (min); (10) reperfusion duration (h); (11) induction anesthetic; (12) measurement of IS; (13) measurement of variance; (14) control group sample size; (15) control group mean infarct size; (16) control group variance; (17) conditioning group sample size; (18) conditioning group mean infarct size; (19) conditioning group variance

### Meta-analysis

**Fig. 2:**
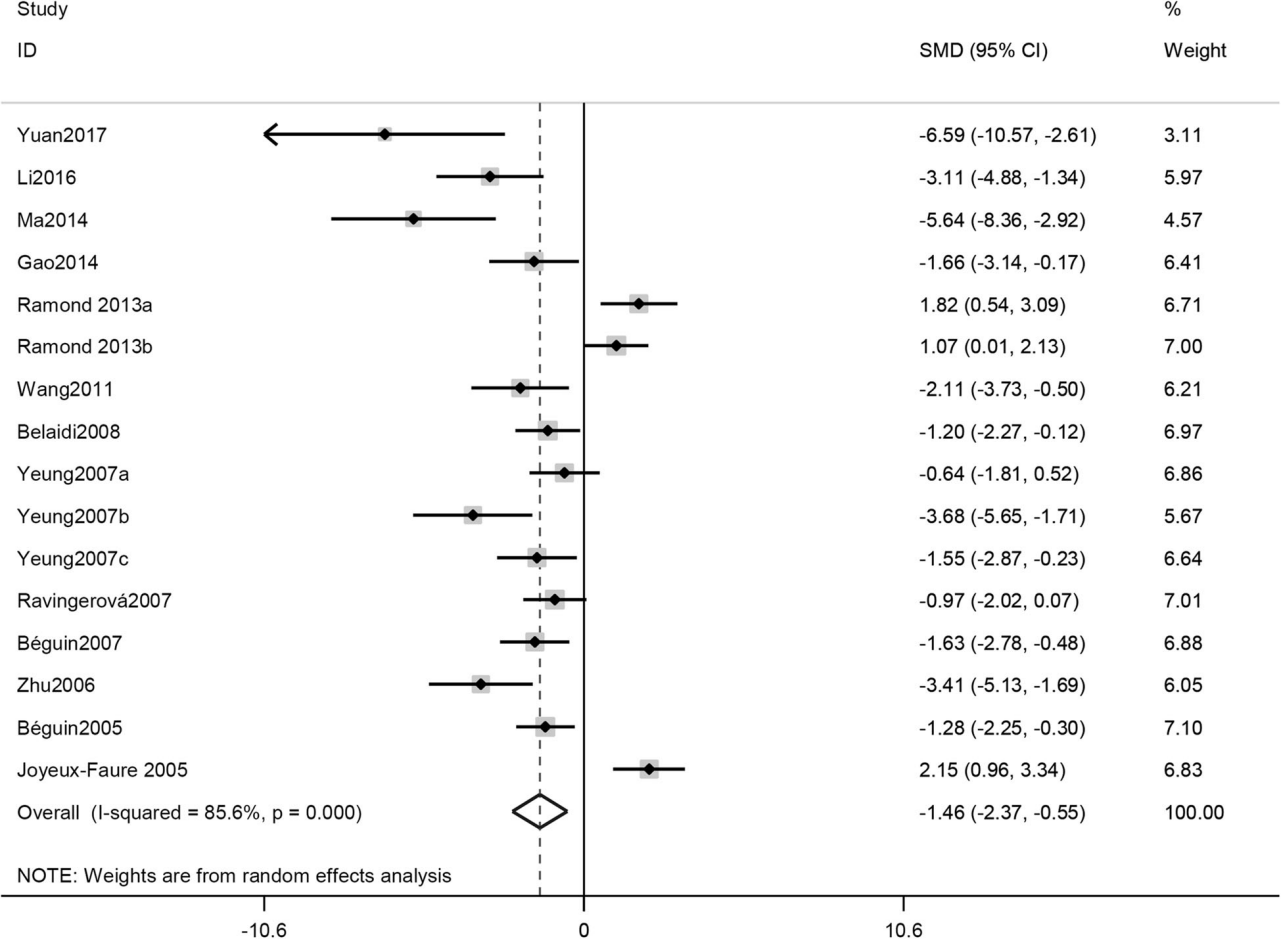
Forest plots of the effect of IH on IS/VS or IS/AAR% in the in vitro group pooled using random-effects meta-analysis

**Fig. 3:**
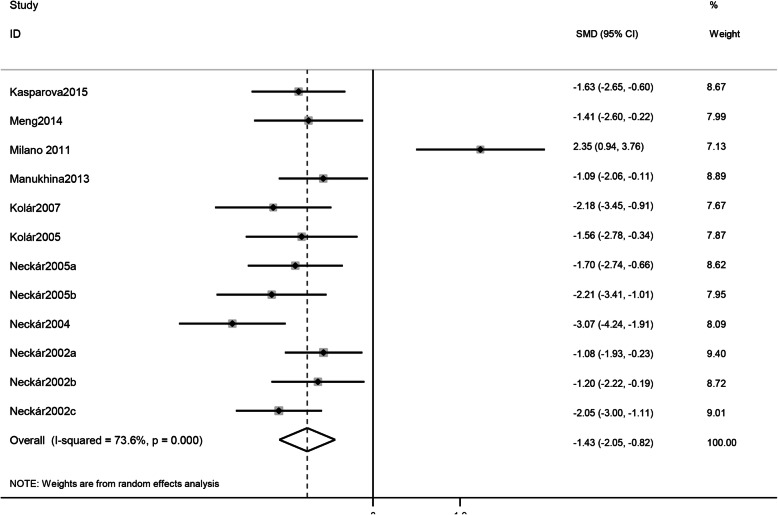
Forest plots of the effect of IH on IS/VS or IS/AAR% in the in vivo group pooled using random-effects meta-analysis

The meta-analysis showed that IH reduced IS after AMI both in vitro group (SMD -1.46, 95% CI [− 2.37, − 0.55]; I^2^ = 85.6%, *P* = 0.000; Fig. 2) and in vivo group (SMD -1.43,CI [− 2.05, − 0.82], I^2^ = 73.6%,*P* = 0.000; Fig. 3).

### Sensitivity analysis

**Fig. 4:**
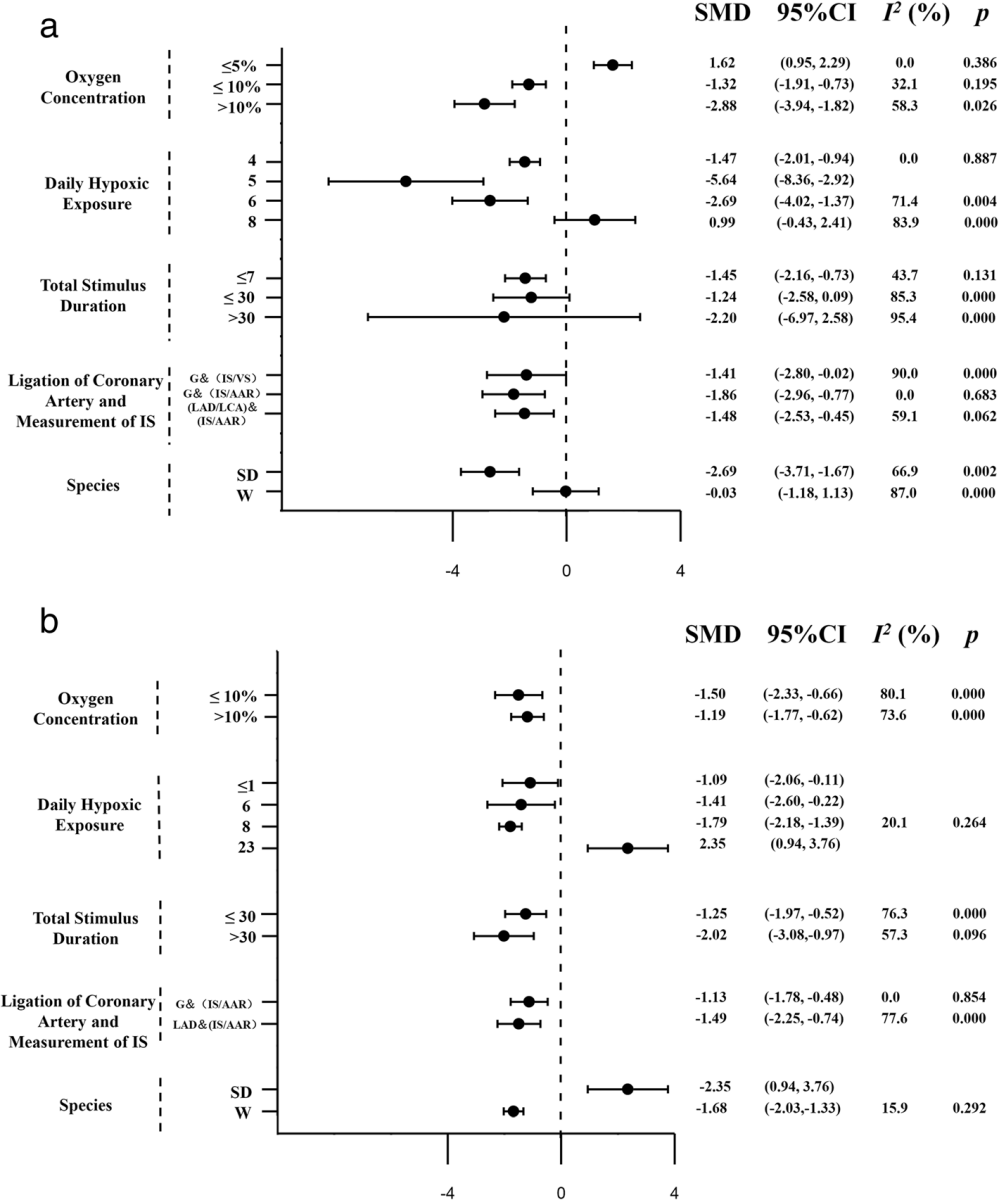
Subgroup analysis of the effect on the vitro group (**a**) and the vivo group (**b**). Experimental variables are used to obtain the weighted standard mean difference along with the corresponding 95% confidence interval (95% CI) followed. However, the reported I^2^ and *P*-value was obtained by Q test, with values I^2^ > 50% or *P* < 0.1 taken to indicate moderate-to-high heterogeneity

WMD was used to rerun the meta-analysis, it showed similar results in vitro group (WMD -9.13, 95% CI [− 14.67, − 3.60], I^2^ = 92.3%, *P* = 0.000) and in vivo group (WMD -14.026, 95% Cl [− 20.39, − 7.67], I^2^ = 85.2%, *P* = 0.000). Subgroup analysis including oxygen concentration, daily hypoxic exposure, total stimulus duration, ligation of coronary artery and measurement of IS, species were made subsequently both in the in vitro group and in the in vivo group. In the in vitro group, data was grouped by the level of oxygen concentration (FiO_2_ ≤ 5, 5% < FiO_2_ ≤ 10%, FiO_2_ > 10%), showed a significant reduction in heterogeneity in each subgroup (Respectively I^2^ = 0%, *P* = 0.386; Group B, I^2^ = 32.1%, *P* = 0.195; Group C, I^2^ = 58.3%, *P* = 0.026;Fig. 4a).

### Quality assessment and risk of bias

**Fig. 5:**
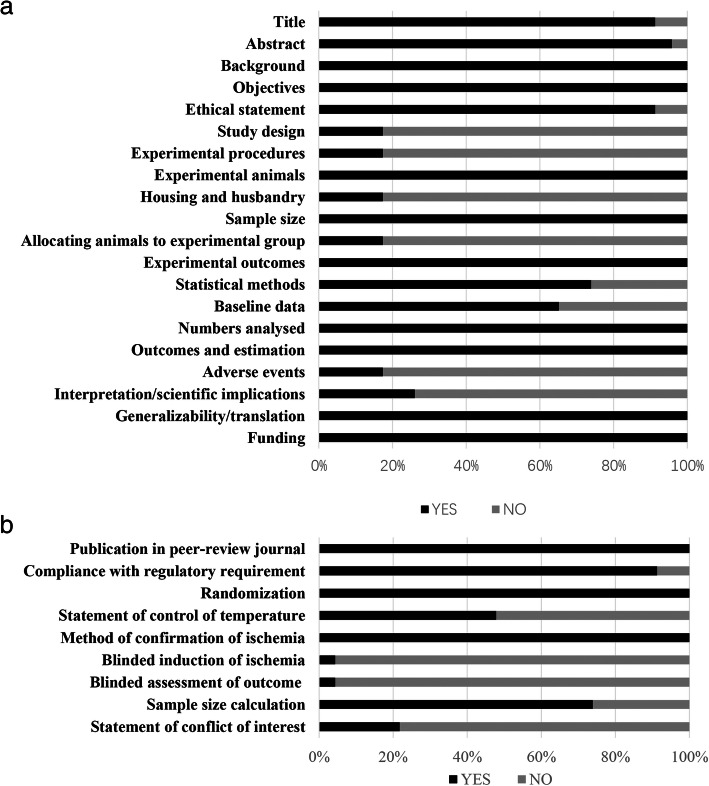
Study quality assessment. A 20-point document quality scoring scale (**a**) based on the ARRIVE guidelines and a 9-point document quality scoring scale (**b**) based on the CAMARADES list. Values are expressed as the percentage of studies reporting each quality indicator

We evaluated the quality of the included literature using a 20-point document quality scoring scale (Fig. 5a) based on the ARRIVE guideline and a 9-point document quality scoring scale (Fig. 5b) based on the CAMARADES list.

**Fig. 6:**
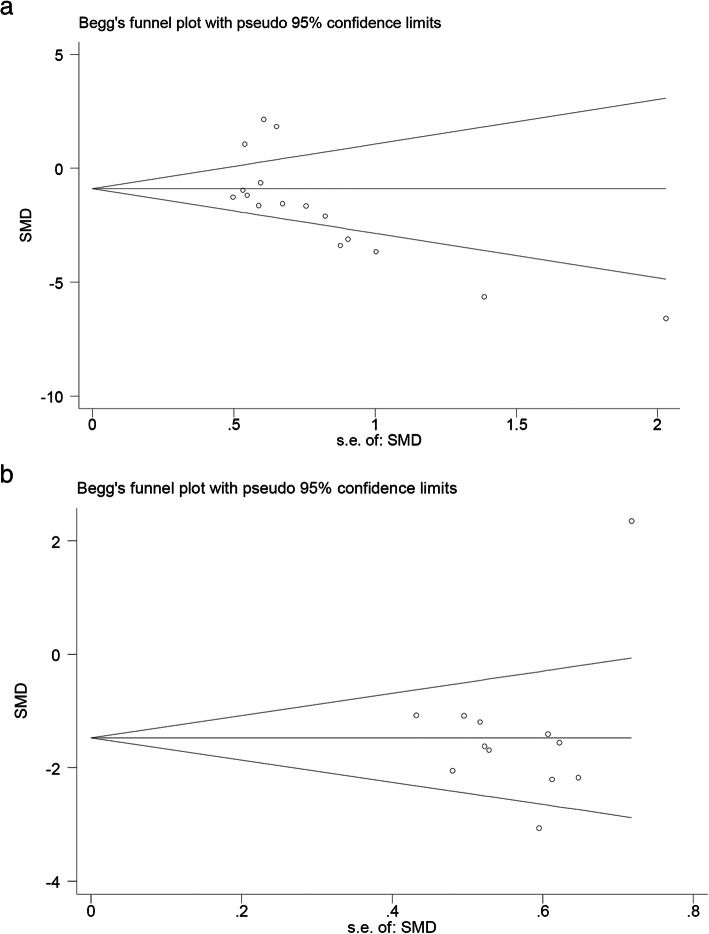
Publication bias detection in the in vitro group (**a**) and in the in vivo group (**b**). Begg’s test is used to help to assess for bias

At the same time, Begg’s test was used to evaluate publication bias. Within the in vitro group, there was significant publication bias (*P* = 0.011, Fig. 6a), and trim-and-fill computation was then used to detect the effect of publication bias on the results. The results showed no changes in the final conclusion. Within the in vivo group, there was no publication bias (*P* = 0.554, Fig. 6b).

## Discussion

The results of this meta-analysis suggest that appropriate IH reduces myocardial infarction size after AMI in rats. Among the conditions of IH, oxygen concentration is one of the most important factors.

The analysis also suggest a high degree of heterogeneity among the included studies; therefore, we conducted further subgroup analyses to identify sources of heterogeneity. We used subgroup analysis to investigate the effects of oxygen concentration, daily exposure time, total days of exposure, ligation of coronary artery and measurement of IS, species on myocardial infarction area after acute hypoxia in rats.

In the in vitro group, hypoxic concentrations ≤5%, ≤10, and > 10% were grouped, and the heterogeneity between the groups was significantly lower than before. Further sensitivity analysis showed that the results were credible. We also performed a subgroup analysis on the ligation of coronary artery and measurement of IS subgroups in the in vitro group, and the results of the G/(IS/VS) group also showed a high degree of heterogeneity. Thus, we conducted a further subgroup analysis of the hypoxia concentration in this subgroup (Supplementary material; Figure 1), and it suggested that the heterogeneity among the subgroups was significantly reduced. Similarly, through analysis of the species of rats in the in vitro group, the results in the Wistar rats group indicated that intermittent hypoxia might not reduce the area of myocardial infarction. Then we conducted a further subgroup analysis of the hypoxia concentration in this subgroup and found that the heterogeneity of each group was significantly reduced (Supplementary material; Figure 2), indicating that oxygen concentration may be an important factor in determining whether intermittent hypoxia can reduce the area of acute myocardial infarction in rats.

Thus, oxygen concentrations may be a source of heterogeneity and one of the main factors affecting the change in myocardial infarction size. Slightly higher hypoxic concentrations are more beneficial, and an excessively low oxygen concentration (FiO_2_ = 5%) increases the IS after myocardial infarction in rats, suggesting that the protective effect of IH on the heart requires appropriate hypoxic concentrations. The lower limit for the oxygen concentration (FiO_2_) may be between 5 and 9.5%, and the exact lower limit needs further experimental research.

The subgroup analysis of daily hypoxic exposure suggests that daily hypoxia of 4 h, 6 h, and 8 h can reduce the area of myocardial infarction after acute myocardial infarction in rats. Notably, the protective effect of intermittent hypoxia on the heart is also observed with less than 1 h of daily hypoxia, and daily hypoxia for 23 h may increase the area of myocardial infarction.

Additionally, within the in vitro group, subgroups were defined on the basis of total duration (≤7 days, ≤ 30 days, and > 30 days); the subgroup with a total duration ≤1 week showed a reduction in IS after myocardial infarction. Does this result suggest that short-term hypoxia, perhaps even a single day of hypoxic treatment, can protect the heart? Further research is needed to confirm this conclusion. Short-term hypoxia, if effective, will facilitate the clinical application of IH for AMI and improve patient compliance compared to long-term protocols.

Above all, different hypoxic conditions have different effects on the heart. To clearly propose the treatment standard for intermittent hypoxia in the future, the concept of hypoxic dose which is the result of multiplying the oxygen concentration, daily exposure time, and total number of days should be introduced. However, the current researches are too deficient to clarify the hypoxic dose. Therefore, future research should explore the beneficial range of oxygen concentrations, daily durations, and total days, and then clarify the optimal dose of IH. Researchers must also pay attention to the effect of altitude (atmospheric pressure) and mode of action. Furthermore, the protective effect of intermittent hypoxia on the heart in rats disappeared after 90 days of recovery in a normal oxygen concentration environment [40], therefore, the effective course of hypoxic treatment should be clarified. Moreover, IH can be seen as a specific method of ischemic preconditioning of the myocardium [43], which reduces ischemia-reperfusion injury through a variety of cytokines and signaling pathways, further researches are needed to clarify the mechanism.

## Document quality and publication bias

We also assessed the quality and publication bias of the research literature included in this analysis and assessed their impact on the final outcome. Most of the blinded ischemic and blinded results in the experimental procedure were not clearly described, which may increase the estimated IS after myocardial infarction in rats, thereby increasing the effect size. Additionally, descriptions of factors such as experimental design, rat feeding, rat allocation, and adverse events were described with little detail, if at all, and it was difficult to determine whether some of the lower-quality studies strictly applied high-quality experimental design. These factors may be the source of this heterogeneity in the analysis. Begg’s test showed that significant publication bias was present within the in vitro group. The source of this publication bias may be that some negative results were not published.

## Limitations

Since estrogen can potentially protect or damage the heart [44–46], the subjects we included were male rats. In addition, age may also have an effect on the infarct size after acute myocardial infarction in rats. However, there are too few studies using newborn rats as experimental subjects, and more intermittent hypoxia and age-related studies are expected. This systematic review uses inclusion and exclusion criteria for literature screening but does not include articles published in other languages or unpublished articles in any language. There were also a small number of documents for which we failed to find the full text; these documents were excluded.

## Conclusion

This systematic review showed that IH can reduce the infarct size after AMI in rats. Despite the high degree of heterogeneity, sensitivity analysis confirmed that the results were reliable, and hypoxia concentration may be one of the most important factors. We expect that IH will be an effective adjuvant treatment for patients with coronary heart disease and can improve systemic organ function. Future research should further explore the mechanism of action of IH, identify the optimal doses of IH suitable for various organs and tissues throughout the body, include large mammals and animals with comorbidities, and, finally, guide the application of IH in the clinic.

## Supplementary information

**Additional file 1.**

**Additional file 2.**

**Additional file 3.**

## Data Availability

The datasets generated and analyzed during the current study are available from the corresponding author on reasonable request.

## Abbreviations

IH: Intermittent hypoxia
IS: Infarct size
AMI: Acute myocardial infarction
CI: Confidence interval
VS: Ventricle size
AAR: Area at risk
