# Supplementary figures and images for: Intermittent hypoxia reduces infarct size in rats with acute myocardial infarction: a systematic review and meta-analysis
Source: BMC Cardiovasc Disord. 2020 Sep 22;20:422. doi: 10.1186/s12872-020-01702-y (PMC7507284; doi:10.1186/s12872-020-01702-y)

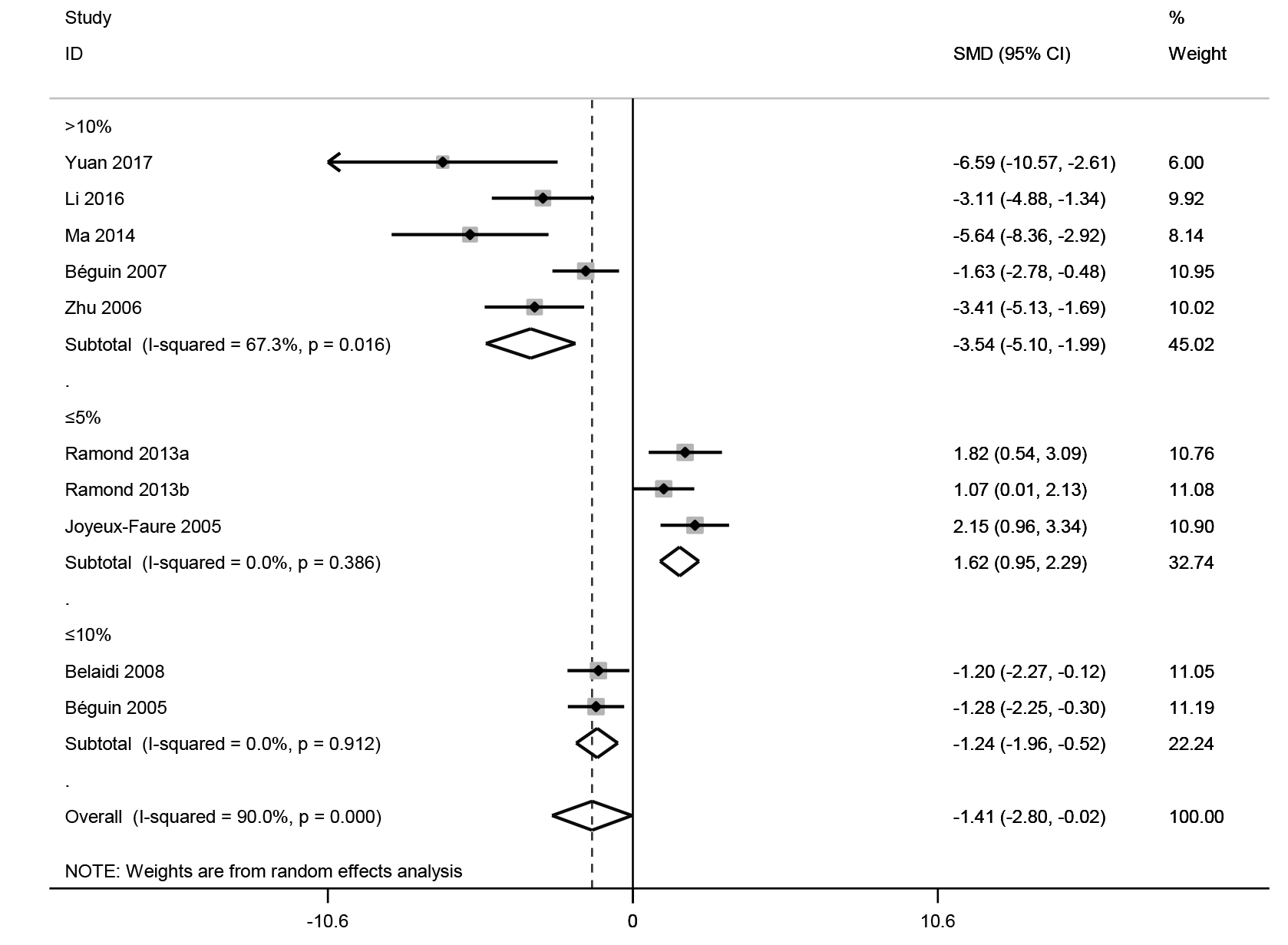

Supplement: Supplementary file 1 — Additional file 1. [file 12872_2020_1702_MOESM1_ESM.tif]

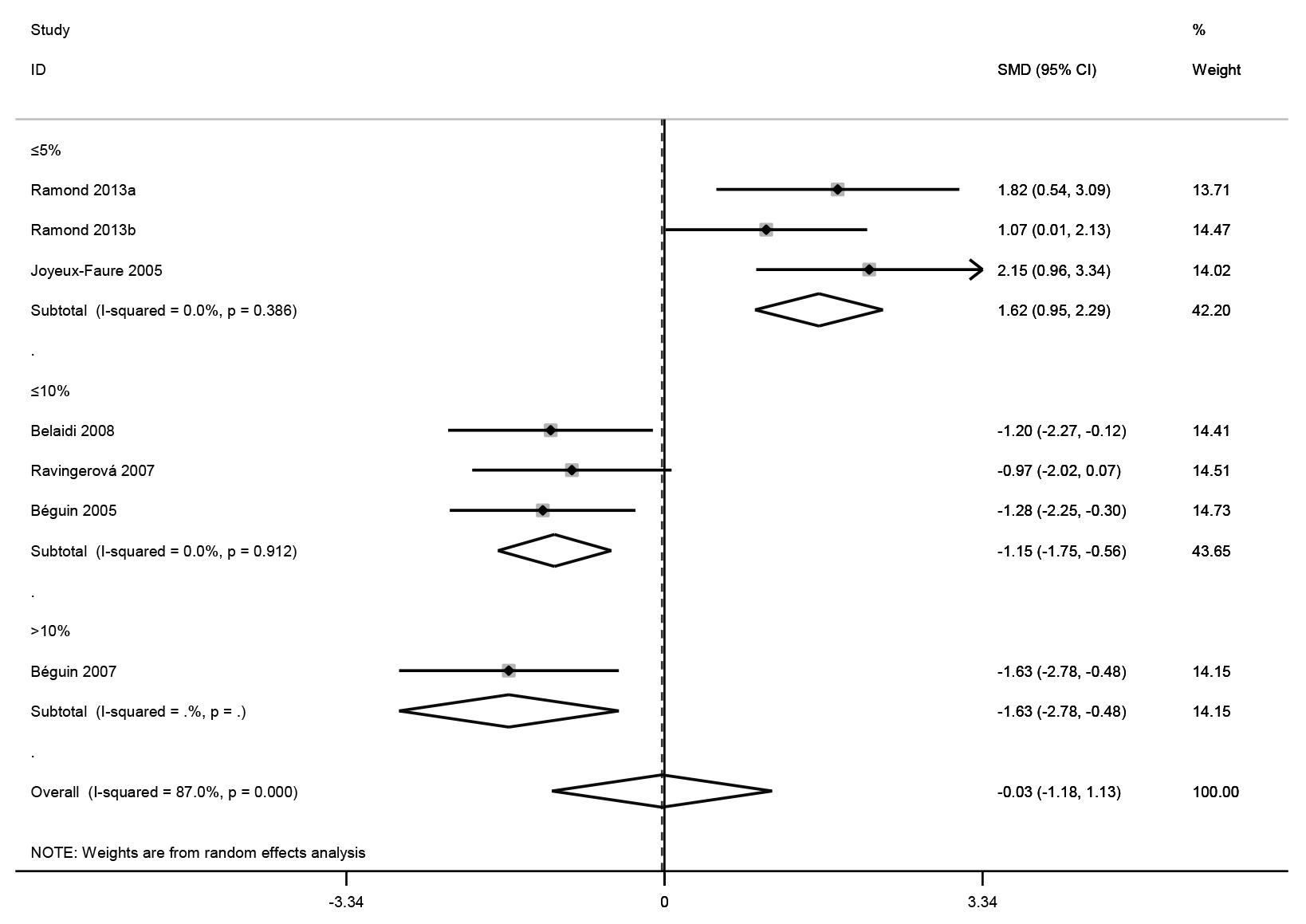

Supplement: Supplementary file 2 — Additional file 2. [file 12872_2020_1702_MOESM2_ESM.tif]
