# Supplementary material for: Intermittent hypoxia reduces infarct size in rats with acute myocardial infarction: a systematic review and meta-analysis
Source: BMC Cardiovasc Disord. 2020 Sep 22;20:422. doi: 10.1186/s12872-020-01702-y (PMC7507284; doi:10.1186/s12872-020-01702-y)
Supplement: Supplementary file 3 — Additional file 3. [file 12872_2020_1702_MOESM3_ESM.pdf]

Study	A 20-point document quality scoring scale																				A 9-point document quality scoring scale								
	1	2	3	4	5	6	7	8	9	10	11	12	13	14	15	16	17	18	19	20	1	2	3	4	5	6	7	8	9
Yuan 2017(4)	+	+	+	+	+	−	−	+	−	+	−	+	+	+	+	+	−	−	+	+	+	+	+	−	+	−	−	+	+
Li 2016(25)	+	+	+	+	+	−	−	+	−	+	−	+	−	+	+	+	−	−	+	+	+	+	+	+	+	−	−	−	+
Kasparova 2015(3)	+	+	+	+	+	−	+	+	−	+	−	+	+	+	+	+	−	−	+	+	+	+	+	+	+	−	−	+	+
Meng 2014(26)	+	+	+	+	+	−	−	+	−	+	−	+	−	−	+	+	−	−	+	+	+	+	+	+	+	−	−	−	−
Ma 2014(27)	+	+	+	+	+	−	−	+	−	+	−	+	−	−	+	+	−	−	+	+	+	+	+	−	+	−	−	−	−
Gao 2014(28)	+	+	+	+	+	−	−	+	−	+	−	+	−	−	+	+	−	+	+	+	+	+	+	+	+	−	−	−	+
Manukhina 2013(29)	+	+	+	+	+	−	−	+	−	+	−	+	+	+	+	+	−	−	+	+	+	+	+	−	+	+	+	+	−
Ramond 2013(17)	+	+	+	+	+	+	−	+	−	+	+	+	+	+	+	+	−	−	+	+	+	+	+	−	+	−	−	+	−
Milano 2011(18)	+	+	+	+	+	−	−	+	−	+	−	+	+	+	+	+	+	−	+	+	+	+	+	−	+	−	−	+	−
Wang 2011(30)	+	+	+	+	+	−	−	+	−	+	−	+	+	+	+	+	−	−	+	+	+	+	+	−	+	−	−	+	+
Belaidi 2008(31)	+	+	+	+	+	+	+	+	+	+	+	+	+	−	+	+	+	−	+	+	+	+	+	+	+	−	−	+	−
Yeung 2007(32)	+	+	+	+	+	−	+	+	−	+	−	+	−	+	+	+	−	−	+	+	+	+	+	+	+	−	−	−	−
Ravingerová 2007(33)	+	+	+	+	+	−	−	+	−	+	−	+	+	+	+	+	−	−	+	+	+	+	+	+	+	−	−	+	−
Kolár 2007(34)	+	+	+	+	+	−	−	+	−	+	−	+	+	+	+	+	−	+	+	+	+	+	+	−	+	−	−	+	−
Béguin 2007(35)	+	+	+	+	+	+	−	+	+	+	+	+	+	−	+	+	−	+	+	+	+	+	+	+	+	−	−	+	−
Zhu 2006(36)	+	−	+	+	−	−	−	+	−	+	−	+	+	−	+	+	−	−	+	+	+	−	+	+	+	−	−	+	−
Kolár 2005(37)	+	+	+	+	−	−	−	+	−	+	−	+	+	−	+	+	−	−	+	+	+	−	+	−	+	−	−	+	−
Béguin 2005(38)	+	+	+	+	+	+	+	+	+	+	+	+	+	−	+	+	+	+	+	+	+	+	+	+	+	−	−	+	−
Joyeux-Faure 2005(39)	+	+	+	+	+	−	−	+	+	+	−	+	+	+	+	+	−	−	+	+	+	+	+	+	+	−	−	+	−
Neckár 2005(40)	+	+	+	+	+	−	−	+	−	+	−	+	+	+	+	+	+	+	+	+	+	+	+	−	+	−	−	+	−
Neckár 2004(41)	−	+	+	+	+	−	−	+	−	+	−	+	+	+	+	+	−	−	+	+	+	+	+	−	+	−	−	+	−
Neckár 2002(42)	+	+	+	+	+	−	−	+	−	+	−	+	−	+	+	+	−	−	+	+	+	+	+	−	+	−	−	−	−
Neckár 2002(43)	−	+	+	+	+	−	−	+	−	+	−	+	+	+	+	+	−	+	+	+	+	+	+	−	+	−	−	+	−
